# Increasing and sustaining diabetic retinopathy screening in Fiji by leveraging community health workers (CHWs) services: A qualitative study

**DOI:** 10.1016/j.heliyon.2022.e11379

**Published:** 2022-11-04

**Authors:** Sharan Ram, Masoud Mohammadnezhad, Komal Ram, Kirti Prasad, Moneeta Pal, Prarthana Dalmia

**Affiliations:** a Center for Public Health Research, Massey University, Wellington, New Zealand; b School of Nursing and Healthcare, University of Bradford, Bradford, UK; c The Fred Hollows Foundation New Zealand, Auckland, New Zealand

**Keywords:** Inequity, Community health workers, Fiji, Diabetic retinopathy, Zone nurses

## Abstract

**Introduction:**

Inequities in access to diabetic retinopathy (DR) services particularly in rural and remote Fiji is concerning. This is because DR when left undiagnosed and untreated for long, can lead to vision loss and permanent blindness. Appropriate channels must be explored to strengthen services and ensure equitable access to healthcare for everyone. This study describes the development and implementation of DR awareness training for community health workers (CHWs) and their subsequent engagement to raise awareness and scale-up DR screening for communities throughout Fiji.

**Materials and method:**

As part of a programme to reduce the incidence of avoidable blindness due to diabetes amongst people living in the Pacific, DR training for primary level nurses was developed and implemented. As these primary level nurses were already inundated by clinical duties and competing health priorities, a shifting of the task was proposed to engage the CHWs who would instead educate communities on diabetes and DR and make referrals for DR screening. A one-day DR awareness training was developed and implemented by the Pacific Eye Institute with funding from the Fred Hollows Foundation New Zealand.

**Results:**

At the end of the DR programme in 2019, the team had achieved their target and trained a total of 823 CHWs giving an 81.32% coverage of the total 1012 registered CHW in the MHMS register. Anecdotal evidence showed a spike in DR referrals and screenings recorded at health facilities. Three key themes emerged related to the involvement of CHWs which include engagement of CHWs, benefits of the engagement, and health system-related challenges.

**Conclusion:**

The use of CHWs who are already integrated into the health system was considered a sustainable intervention to strengthen diabetes and DR services at the primary level of care, particularly if it involves community awareness, health education, and health services facilitation The future of the CHWs will depend on their being integrated more systematically into local health services with strengthened management and supervision.

## 1 Introduction

Diabetes mellitus (DM) is one of the major public health challenges of the 21st century. The International Diabetes Federation (IDF) has estimated that DM currently affects over 463 million people in the world and is expected to increase to 200 million in 2045 [1]. The top five countries in the world with the highest prevalence of diabetes among those aged 20–79 years are all from the Pacific, led by Tokelau with a prevalence of 37.5% [2]. Seven Pacific Island Countries (PICs) are in the top ten worldwide for the prevalence of diabetes and are estimated to remain so by 2035 [2]. A study by Brian, Ramke, Maher, Page, Szetu [3] found that the prevalence rate of diabetes, adjusted for ethnicity, age, and gender, in Fijians aged 40 years or older was 41%. Approximately 60% of all diabetics in Fiji are undiagnosed. Inequities in terms of geographic location have been identified in Fiji, with those living in rural areas less likely to have their diabetes diagnosed [3]. This comes with an anticipated increase in the prevalence of diabetic retinopathy (DR), a complication of diabetes that affects the eyes. Every diabetes patient is at risk of developing DR, which when left unmanaged, can lead to vision loss and permanent blindness. The high number of potentially undiagnosed DR cases and the outstanding barriers to DR management include a lack of appropriate resources in primary health centres (HC), and primary level clinicians and community health workers (CHWs) limited knowledge of DR screening, diagnosis, and management [4].

To alleviate these issues and address diabetes in its entirety, it is important to strengthen the primary and secondary prevention pathways which identify the disease early, halt its progress, and prevent complications [5]. The Ministry of Health and Medical Services (MHMS) in Fiji recognises the important role of CHWs in enhancing primary health care and strengthening referral systems across the levels of health care. In 2015, MHMS launched the CHW policy and manuals to advocate for the critical role of CHWs in Fiji [6]. In line with this, The Fred Hollows Foundation NZ (FHFNZ) partnered with the MHMS and Pacific Eye Institute (PEI) to support DR awareness training for CHWs across the country.

FHFNZ is a not-for-profit, charitable organisation that works towards reducing avoidable blindness and vision impairment in the Pacific. FHFNZ works in partnership with the MHMS, Fiji to support the training of eye health practitioners so that this local eye care workforce can deliver eye care services at PEI in Suva (Central Division), Fiji, on outreach throughout the country and across the Pacific. The goal of the programme “Tackling DR in the Pacific” is to reduce the incidence of avoidable blindness due to diabetes amongst people living in the Pacific. The overarching aims of the Pacific DR Model are to “Prevent DR”, “Treat DR” and “Understand DR”. These aims guide the diverse range of programme activities, making it a multi-pronged approach to address DR in its entirety [7]. It is important to strengthen the primary and secondary prevention pathways which identify diabetes and DR early, halt its progress, and prevent complications. FHFNZ in partnership with the Queen Elizabeth Diamond Jubilee Trust, MHMSFiji, and PEI has supported the DR awareness training for primary level clinicians and CHWs across the country. This training is an important mechanism to increase awareness about diabetes and DR, integrate DR services within the broader diabetes sector, strengthen referral pathways, and streamline care for patients, and improve integration across services. In Fiji, rural health facilities such as health centers and nursing stations are managed single-handedly by Zone Nurses having larger population catchment areas and thus spend more clinical time leaving inadequate time for health education and promotion. As CHWs are part of the communities, it is easier to leverage them for such tasks with adequate capacity building and empowerment.

This study describes how the training of zone nurses was shifted to engage CHWs to scale up DR awareness and referrals for screening in the Fijian communities.

## 2 Materials and Methods

### 2.1 Study design and settings

This was a qualitative study and primarily involved semi-structured interviews with Zone Nurses and other key stakeholders (staff of PEI & FHFNZ) from the implementing agencies. The study was conducted in Western, Northern, and Central Divisions in Fiji. The data collection was undertaken in January 2021.

### 2.2 Study participants

A total of 9 key informants were purposively selected to participate in the interviews as these individuals represented the implementing agencies. These included Zone Nurses (5) who manage the CHWs, trainers from PEI (2), and the funding entity which is FHFNZ (2). All the participants were identified by the DR Coordinator.

### 2.3 Interventions for CHWs (DR awareness training)

The capacity strengthening training to create awareness and promote DR screening and treatment amongst MHMS primary health staff in Fiji began in September 2015 in the Central Division. For DR awareness training, the target group was primary health clinicians such as the district nurses who worked alone in the Nursing Stations, the Zone Nurses, and Special Outpatients Department (SOPD) nurses in HCs. The SOPD is the entry point of diabetes patients into the health system for review. These Zone Nurses are trained with the view to strengthen the domiciliary care in each of the nursing districts, especially the follow-up of the diabetes cases for eye screening. The Zone Nurses are after all strategically positioned closest to the communities as frontline workers and believe that with some training - they would be ready for the challenge. One year on, the programme was extended to the 2 other administrative divisions of Fiji: Western and Northern. The DR awareness training programme was new amongst primary health clinicians, such as nurses, and gained momentum quickly in Fiji. However, as the Zone Nurses are swamped with clinical duties, CHWs were instead proposed to undergo the DR awareness training who would educate communities on DR and make referrals to HF. The DR awareness training for Zone Nurses was thus re-designed to make it more suitable for the CHWs with the contents of the training described in Table 1.

**Table 1:** Contents of DR awareness training for CHWs.

Sections & Topics	Subtopics
SECTION ONE: What is diabetes	Types of diabetes
	Major risk factors
	Signs and symptoms
	Complication screening
	Preventative methods and early detection screening
	Key factors in control and management of diabetes.
	Case Study One
SECTION TWO: What is Diabetic retinopathy?	The back of the eye
	Functions of the retina
	What is diabetic retinopathy?
	Can DR be prevented?
	Controlling the risk factors
	Can it be treated?
	Preventative methods and early detection screening
	Suggested health education activities
	Case Study Two
SECTION THREE: Referral and Data Collection	Diabetic Eye Referrals
	Data Collection about eye screening in your area

### 2.4 Data collection tools

A semi-structured open-ended interview guide was developed after an extensive review of local and international literature. The guide attempted to capture views of various stakeholders on the need for task-sharing and the design of the training DR awareness programme. The guide contained questions on the benefits of training and the role of CHWs in strengthening links between community and health system, training gaps, the need to extend the training to CHWs, health system-related challenges, and strengthening referral pathways.

### 2.5 Data collection

Once the potential participants were identified, they were verbally explained the rationale of the study including study methods over the phone using the Participant Information Sheet (PIS) by the principal investigator. Semi-structured interviews were conducted with the Zone Nurses face to face whilst the same was done via video conferencing using Microsoft Teams for participants not available to meet in person. Participants were provided with a copy of PIS which explained the purpose of the study along with the consent forms. Those agreeing to participate were required to return the signed consent forms.

For each interview, a suitable time convenient to both the interviewer and the participant was agreed upon and locked (by both the participants and the researcher) to convene the data collection online using Microsoft Teams.

At the outset, the researcher reassured the participant that the information shared by them would be confidential and anonymous and that participants could withdraw from the study at any time without any consequences. Participant permission was also sought to audio record the sessions. The researcher facilitated the interviews, and all interviews were conducted in the English language. Each interview took approximately 40 min. No compensations were provided for participants of the interviews.

### 2.6 Data analysis

The interviews were audio-recorded and transcribed verbatim. The data were analysed using thematic analysis techniques [8]. A general inductive approach was utilised for analysing the collated qualitative data [9]. Emerging themes from the data were identified and adjudicated before finalising the themes.

### 2.7 Ethical approval

The ethics approval for the study was obtained from the Fiji National Health Research Ethics Committee (FNHREC) of the Fiji MHMS (ID 303.20).

## 3 Results

The survey findings can be grouped into three themes including engagement of CHWs of DR awareness training, benefits of leveraging the CHWs, and health system-related challenges.

In the analysis that follows, indicative commentaries from the participants are inserted to illustrate the views held by them. Quotations are presented based on the participant's expertise. For Zone Nurses, they are assigned initials ZN, IA refers to implementing agency which is PEI and FA refers to funding agency which is FHFNZ.

### 3.1 Theme 1 engagement of DR awareness training for CHWs

The DR awareness training for community health nurses (CHNs) was paused. Implementers proposed that to have a better reach of the communities, there was a need to empower the CHWs to reach the communities effectively and thus shift training geared for CHNs to CHWs and was planned for the following year which was 2017.

“When we were midway into the training of CHNs on DR, the nurses proposed to involve the CHWs as they lived in communities and registered with MHMS and so that they could be free up for clinical work and it was easy enough talk for CHWs to educate the community and refer for screening. The proposal was taken to the funding agency. They had to re-allocated on budget as it meant more training and the content re-designed to focus on training CHWs”

FA 01

As the capacity strengthening training activity was not part of the initial approved Project Design Paper, additional funding was sought to train CHWs. This was the beginning of the DR awareness training for CHWs.

“The reason to establish the CHW training was to ensure that we strengthen the patient referral pathways to ensure that we are able to reach more and more people, not only about diabetes but also referring people with DR for screening. It was to make them aware that if they have diabetes, then they are at risk of getting DR as DR is asymptomatic and often gets undiagnosed. People are unable to link DR to diabetes so we wanted to address these challenges and so the CHWs were identified as the key people and first point of contact for the community who could tell patients about DR and refer them for screening.”

FA 02

In 2018, the Fiji DR programme acknowledged the provision of more funding to go ahead with the DR awareness training for CHWs. At the end of the DR programme in 2019, the team had achieved their target and trained a total of 823 CHWs giving an 81.32% coverage of the total 1012 registered CHW in the MHMS Register.

“Having worked in those roles for a long time, I’ve seen the big difference because when we just started, we were just concentrating on patients that were coming by themselves, and then after a while, we became stagnant, we were just seeing the same patients all over again. When CHWs were trained at lower levels of care, things drastically changed for the better, people started to come because the CHWs were empowered with the new knowledge and they have different attitudes now and they changed their behaviour to make sure that every diabetic had an eye care examination, so that has made a huge difference.”

IA 02

CHW has implemented a more aggressive strategy in case finding by way of visiting all houses to find any potential case(s) that needs to be screened for DR.

“CHWs have been going door to door after the DR awareness training to educate people on diabetes and DR. They carry the referral forms with them to refer patients for DR screening as well as for referrals for other diseases.”

ZN 01

### 3.2 Theme 2 benefits of leveraging the CHWs

According to the IAs, several benefits was noted by engaging the CHWs since the implementation of the DR awareness training for CHWs. These included improved profiling by CHW, an increase in the numbers of eye referrals as well an increase in the number attending SOPD and non-communicable disease (NCD) screening programs.

“We’ve been and as a receiving feedback from the HF staff that there have been a lot more people coming for DR screening and these have been referred by CHWs.”

IA 03

The Zone Nurses described that training CHWs in DR awareness was valuable as the health systems considered the CHWs as the eyes and ears of the community as they are strategically positioned in the communities.

“CHWs have grown to be part of our team and give us a call when they feel the need for home visits, for instance when someone is sick or for dressing. They are our eyes and ears in the community. They are essential because one Zone Nurse looks after over 3000 people in an area or more than 10,000 people in the community and so we don’t know every one of them whilst the CHWs know all about them”

ZN 03

Zone Nurses enjoyed a good relationship with the CHWs. Zone Nurses trusted the CHWs, and it was a way to get the insider's view of the community in terms of the health status of the community.

“We appreciate the work of CHWs and they are doing a fabulous job by giving information on their patients and keeping the record. They have been very helpful with community outreach.”

ZN04

The Zone Nurses had a lot of appreciation for the CHWs and mentioned that CHWs worked above their expectations compared to what they were paid. They felt that there was a need to continually train the CHWs as they build experience and have better community liaison and leadership skills.

“CHWs are like our eyes and ears in the community. As Zone Nurses, we look after hundreds of people in a particular catchment area and so we don’t know every person whilst the CHWs know all of them. They also feed us information about village health and if we need to attend to some cases in the villages especially when someone is very sick.”

ZN 01

The Zone Nurses also acknowledged the liaison role that CHWs played because they assisted the Zone Nurses by being in the community and served as a link and entry point into the community.

“Whenever we have to do outreach, we just reach out to CHWs. They disseminate the information in the village and also liaise with the village chief so all the formalities are sorted and we can then just go and carry out our screening work”

ZN 01

Zone Nurses are already drowned in their clinical duties, and as such their workload was reduced with the help of CHWs. They mentioned receiving help in logistics during outreach planning and that they were committed to the work they did and communicated, when necessary, to bring their attention to the health aspects of the community.

“CHWs have a flexible schedule. Some of them work for 3 hours per day 3 days per week. When we have outreach visits, they work with us in planning, informing the community, and getting the people for screening. They also make requests for outreach when they feel the need for it. They work a lot more than what they are paid. Some of them work hard to serve their communities.”

ZN 04

Further, as CHWs are based within their communities and volunteered to take up this position, they are passionate about their work and a lot of them spend more time serving their communities beyond the number of hours they are required. CHWs were paid a monthly honorarium allows of $200 Fijian dollars.

### 3.3 Theme 3 health system-related challenges

The Zone Nurses shared some challenges with the programmes involving CHWs related to the feedback system and outreach clinic. Nurses felt that there was some broken chain in terms of the feedback provided to the CHWs in terms of referral. According to the Zone Nurses, CHWs should be informed whether the patient whom they referred to the Health Facility (HF) has been attended to because not all patients end up accessing the services for one reason or another.

“When CHWs refer a patient to HF for screening, we are unable to inform them whether their patients have been screened or not. We can only do that when we meet them once a month. This way there is a chance to lose a patient and CHWs would not be able to follow up promptly.”

ZN 03

As a follow-up, a clear feedback system such as a Viber™ chat platform used by some groups is useful. Unfortunately, this will not work for where internet connectivity is an issue.

“If CHWs send a patient, then we have to inform them. We need a better feedback system so that they (CHWs) know that their patients have been seen. A lot of times the patient can lie about coming to the hospital despite them having the referral form. That is the feedback we have also received from some CHWs.”

ZN 02

This affects patient follow-up, particularly of patients who are most at risk of developing adverse eye health outcomes.

Another challenge Zone Nurses faced was that for communities close to the hospital, fewer people turned up for outreach services because they felt that the hospital was near, and they would just go to the hospital if they needed any services.

“People from the communities close to townships did not usually turn up at outreach because they think they that they can go to the hospital because the hospital is nearby.”

ZN 05

At the national level, when there are other priorities, the CHNs are pulled to focus on the immediate or key priorities at a particular point in time. This shifts the focus of Zone Nurses and thus communities are susceptible to neglect. However, when this take is delegated to CHWs, there is continuity in community education, advocacy, and referrals to HF.

“In the past, training for nurses on DR came to halt because nurses are required to change focus to other health campaigns as the need arises or as per the national priorities for instance during the National Men-C Vaccination campaign where all community health nurses were involved to cover children between the age 1–19 years by September 2018 and the Micro-filarial density Campaign”

IA 01

According to the IA, at the macro level, the most challenging aspect of the program implementation is its total dependency on the MOH&MS stakeholders. This meant that IA and FA had to ensure that the communication lines to the managers and supervisors were always open, clear, and transparent to gain support and involvement, especially with the training.

## 4 Discussion

Fiji has been experiencing a growing burden of NCDs, particularly diabetes [10], posing a significant threat to the existing weak health care system in the country. Further, the inequity in access to health services is exacerbated by rural and remote locations, high levels of poverty, and a lack of social support and security. In this context, this study outlined the engagement of CHWs to educate communities about DR, strengthen referral pathways, increase the uptake of DR screenings, and in turn, improve outcomes for eye health. It also outlines the development and implementation of a training program whereby upon adequate deliberation among their key groups namely the Zone Nurses, the implementing (PEI), and the funding agency (FHFNZ), the CHWs participated in one-day DR awareness training. They were provided the required support to educate their communities, identify patients, and refer them for screening of DR.

The findings showed that there were several benefits of leveraging the CHWs in this endeavor. The benefits for the communities included increased knowledge of the eye, eye health, diet, risk of DR among diabetics, and availability of DR services. In the current study, the Zone Nurses and implementers proposed the use of CHWs for advocating for DR and referrals for screening. An increase in screening referrals of patients to the HF was noted through the registers at the HFs which is thought to reflect the aggressive DR advocacy work by the CHWs. In Fiji, CHWs have been engaged in several initiatives for over three decades ranging from maternal child health, water and sanitation, disaster preparedness, and non-communicable diseases [11]. Their critical role in their communities and significant contribution has been acknowledged as the CHW cadre is now formalised through registration within the health system, where a 3-year agreement with the MHMS is signed, and the role of CHWs has been better integrated into the health system.

Due to the lack of awareness of other complications of diabetes, HFs continued to see the same patients over time in their SOPDs in their respective catchment areas, particularly for reviews. New patients have not been forthcoming. The findings of this study suggest that the CHWs have been effective in both advocating about diabetes and its other complications and making referrals for screening.

Previous studies have shown that DR services were often not accessed due to a myriad of reasons, such as lack of public awareness, lack of resource allocation for diabetes and its complications, and high transportation costs to access services in main centers, and thus, those in rural and remote areas were at higher risk of DR [12, 13]. In particular, the delay in the diagnosis of diabetes, as well as poor glycemic control, resulted in severe DR in Fiji [14]. Lack of uptake in screening necessitated a change in approach that is from engaging the Zone Nurses to CHWs to reach the rural and remote communities more effectively. The impact of DR awareness training found that the CHWs’ knowledge improved and referrals had significantly increased [15]. Apart from engaging the CHWs already integrated into the Fijian health care workforce, a second benefit was that the CHWs spoke the local language and are adapted to the cultures of the community, and are already engaged in the community also demonstrated by Callan, Sundin, Suffian, Mehta [16]. Several studies have shown that engaging CHWs reduces the pressure on nurses who can focus on the clinical aspects of the job as well as reduces costs for the health sector. CHWs also strengthen the links between the patients and the community and social services [17, 18].

Household members who participated in a study on awareness and use of eye care services in Fiji were generally aware of conventional eye care services, however, these services were under-utilised [19]. In general, fatalistic attitudes, being older, rural residence, and female gender were the most common reasons for not seeking conventional eye care services. To improve eye health, any local barriers to eye health care uptake needs to be reduced. It can be inferred that engaging CHWs has overcome several of these barriers. Consistent advocacy, visitation, encouragement; accompanying of domiciliary cases to HFs, and provision of the referral form increased referrals for DR screening [20, 21].

Challenges in engaging CHWs included Zones Nurse's inadequacy to provide feedback to CHWs on whether the individual referred had accessed screening as this determined if CHWs need to follow-up with patients. This is due to inadequate channels of communication. For instance, some CHWs did not own a mobile phone or there is no mobile connectivity in their locality due to remoteness from main centers and providing feedback to CHWs becomes difficult. Additionally, the feedback system is not automated in the sense that CHWs do not receive automatic feedback that patients referred by them have been screened and is a common challenge noted elsewhere [22]. Monthly catch-up meetings of the CHWs and the Zone Nurses are when such feedback is provided. Challenges in record-keeping by CHWs are variable in other settings. More emphasis on proper record keep would be useful in providing an update on community health [23].

Capacity building and empowerment of the CHWs through training, mobilisation, information sharing, and greater integration through dialogues have the potential to enhance utilisation by rural populations [24, 25] and reduce inequitable access to healthcare. In the current context, the CHWs and the Zone Nurses formed a team that worked together to establish and maintain an environment that promoted positive well-being by sharing information, conducting activities, and providing assistance to support the healthcare needs of communities CHWs also provided patients with social support and connected community resources which is yet another benefit of engaging them and is well documented in literature from other settings [22, 26].

### 4.1 Limitations

The present study has a small sample size; however, it is significant in that it comprehensively identifies the views of all stakeholders on the need for shifting the task of educating communities to CHWs as all communities have a CHW and leaving nurses to focus on clinical duties.

## 5 Conclusion

This study demonstrates that it was beneficial to engage CHWs in increasing DR awareness and strengthening referrals for DR screening. Although this study does not permit triangulation of results, high rates of screening recorded at HFs are suggestive that the DR awareness training has been an impactful strategy to reach various communities. It can therefore be argued that provided communities become more aware of DR and referral pathways are clear, coupled with good DR services, uptake of such services will increase including from rural areas. These are important findings for the ongoing DR awareness training intervention. Future research to solicit the views of the communities would provide a better understanding of the perceptions of communities regarding the work of the CHWs. Anecdotal evidence on the high referral rates by the CHWs is thought as a strong indicator of their success in educating and engaging their communities.

## Declarations

### Author contribution statement

Sharan Ram: Conceived and designed the experiments; Performed the experiments; Analyzed and interpreted the data; Contributed reagents, materials, analysis tools or data; Wrote the paper.

Masoud Mohammadnezhad: Conceived and designed the experiments; Performed the experiments; Analyzed and interpreted the data.

Komal Ram, Kirti Prasad, Moneeta Pal and Prarthana Dalmia: Conceived and designed the experiments.

### Funding statement

This work was supported by Fred Hollows Foundation New Zealand.

### Data availability statement

Data will be made available on request.

### Declaration of interest's statement

The authors declare no conflict of interest.

### Additional information

No additional information is available for this paper.
